# On the Interactions and Synergism between Phases of Carbon–Phosphorus–Titanium Composites Synthetized from Cellulose for the Removal of the Orange-G Dye

**DOI:** 10.3390/ma11091766

**Published:** 2018-09-18

**Authors:** Hesham Hamad, Jesica Castelo-Quibén, Sergio Morales-Torres, Francisco Carrasco-Marín, Agustín F. Pérez-Cadenas, Francisco J. Maldonado-Hódar

**Affiliations:** Carbon Materials Research Group, Department of Inorganic Chemistry, Faculty of Sciences, University of Granada, Avenida de Fuentenueva, s/n. ES18071 Granada, Spain; heshamaterials@hotmail.com (H.H.); jesicacastelo@ugr.es (J.C.-Q.); fmarin@ugr.es (F.C.-M.); afperez@ugr.es (A.F.P.-C.); fjmaldon@ugr.es (F.J.M.-H.)

**Keywords:** microcrystalline cellulose, chemical functionalization, polyphosphates, synergism, physicochemical properties, Orange G, photocatalysis

## Abstract

Carbon–phosphorus–titanium composites (CPT) were synthesized by Ti-impregnation and carbonization of cellulose. Microcrystalline cellulose used as carbon precursor was initially dissolved by phosphoric acid (H_3_PO_4_) to favor the Ti-dispersion and the simultaneous functionalization of the cellulose chains with phosphorus-containing groups, namely phosphates and polyphosphates. These groups interacted with the Ti-precursor during impregnation and determined the interface transformations during carbonization as a function of the Ti-content and carbonization temperature. Amorphous composites with high surface area and mesoporosity were obtained at low Ti-content (Ti:cellulose ratio = 1) and carbonization temperature (500 °C), while in composites with Ti:cellulose ratio = 12 and 800 °C, Ti-particles reacted with the cellulose groups leading to different Ti-crystalline polyphosphates and a marked loss of the porosity. The efficiency of composites in the removal of the Orange G dye in solution by adsorption and photocatalysis was discussed based on their physicochemical properties. These materials were more active than the benchmark TiO_2_ material (Degussa P25), showing a clear synergism between phases.

## 1. Introduction

Environmental catalysis tries to overcome the increasing pollution generated by a progressively more industrialized society through the search and development of novel materials and treatment technologies. Global warming, exponential growing population, intensive agricultural practices, among others, are the major factors affecting the availability of freshwater resources worldwide [1]. Treatment technologies, desalination and reuse of water intend to mitigate water scarcity. In fact, porous and catalytically active materials are continuously developed to be applied in different treatment processes for the removal of organic pollutants in water by adsorption and/or advanced oxidation processes (AOPs). Among others, heterogeneous photocatalysis has demonstrated to be an excellence treatment technology to remove water pollutants by the action of highly reactive oxygen species (e.g., hydroxyl radicals) generated from a semiconductor. In fact, a wide variety of the materials based on oxides (e.g., TiO_2_, ZnO, WO_3_), chalcogenides (e.g., ZnS, CdS, ZnTe, Bi_2_S_3_), nitrides (GaN), phosphides (GaP) and carbides (SiC), as well as free metal semiconductors composed by recent nanostructured carbons, such as graphitic carbon nitride (g-C_3_N_4_) and graphene derivatives, have been applied to different photocatalytic processes [2,3,4,5,6,7,8]. Most of these semiconductors present a limited photocatalytic performance due to a slow transportation of photoelectrons, fast photoelectron-hole recombination, a deficient surface that hinders the redox interaction with reactants and even, metal leaching when are irradiated in water. Thus, expensive and complex binary or ternary combinations of these materials are often proposed [9,10].

TiO_2_ is the most widely applied semiconductor due to a high photo-activity, low cost, relative low toxicity and good chemical and thermal stability [4,11,12]. However, its performance in the visible range is poor so that different strategies, including non-metal and/or metal doping, dye sensitization, coupling semiconductor and the modification of properties such as crystalline phase, crystallite size and shapes and so on, have been studied to improve its photocatalytic efficiency [13,14]. On the other hand, the handling facilities and the price and suitability of the precursor materials should be taken into consideration in the design and development of novel photocatalysts. For instance, photocatalysts are used as building materials and some amounts of them are added to the concrete for the control of indoor air quality, preventing the accumulation of volatile organic compounds (VOCs) on building surfaces by oxidation [15]. Different types of industrial residues were recently reviewed in order to optimize the final price of the photocatalyst [16]. Thus, Ti-photocatalysts were prepared by calcination of the sludge containing Ti-salts previously used in the flocculation of sewages effluents [17] and by using natural phosphates [18]. An interesting approach is the synthesis of Ti-carbon composites [14] due to a better dispersion of Ti-nanoparticles, a well-developed porosity (enhanced pollutants adsorption) and the band gap narrowing by the synergism between phases. The employ of biomass, in particular cellulose [19], as support or carbon source is a remarkable alternative to prepare Ti–carbon photocatalysts, because it is the cheapest and most abundant biopolymer [20].

In this manuscript, carbon–phosphorus–Ti composites were sustainably developed, characterized and used for the photodegradation of Orange G (OG), a typical dye used in the textile industry. Microcrystalline cellulose (MCC) was used as a carbon precursor because is cheap, environmentally friendly and the most abundant renewable material; TiO_2_ was used as semiconductor for the synthesis of nanocomposites. The crystalline structure of MCC required its previous solubilization with an acid treatment before Ti-impregnation, which in turn improved the contact between phases and the dispersion of the active Ti-phase. The influence of the acid pretreatment and the Ti:cellulose ratio on the physicochemical properties of the nanocomposites obtained and on the photocatalytic efficiency of the samples is discussed.

## 2. Materials and Methods

The synthesis of the cellulose–Ti composites was carried out using a procedure reported elsewhere [21]. Briefly, MCC (from Merck, Darmstadt, Germany) was suspended in distilled water (200 g L^−1^) and then, it was completely dissolved by adding 10 mL of phosphoric acid (H_3_PO_4_) under stirring at 50 °C overnight. After that, an appropriated amount of titanium tetra-isopropoxide (TTIP) in heptane was dropped to the previous cellulose solution to obtain different cellulose-Ti composites by changing the corresponding Ti:cellulose mass ratio, namely 1:1, 6:1 or 12:1. The solid suspension formed during the TTIP hydrolysis was aged under continuous stirring at 60 °C for 24 h and then, the composites were filtered, washed with distilled water and acetone and dried at 120 °C in an oven. Finally, the carbon–phosphorus–Ti composites were obtained by carbonization of the corresponding cellulose–Ti composites in a tubular furnace at 500 or 800 °C under 100 cm^3^ min^−1^ N_2_ flow. All samples were grinded and sieved to a particle size of 100–200 μm before used in photocatalysis and characterization. The samples will be labelled as CPTX-Y indicating the composition (C = cellulose, P = phosphoric acid, T = TTIP impregnation), “X” refers the Ti:cellulose ratio used (i.e., 1, 6 or 12) and “Y” states the carbonization temperature (500 or 800 °C). For instance, CPT6-500 corresponds to the composite prepared in a 6:1 ratio and at 500 °C.

The morphology of the materials was studied by scanning electron microscopy (SEM) using an AURIGA Carl Zeiss SMT microscope (Carl Zeiss AG, Oberkochen, Germany). Energy dispersive X-ray (EDX) microanalysis (Carl Zeiss AG, Oberkochen, Germany) was carried out to determine the composition and homogeneity of the samples. This information was completed by analysing the samples with X-ray photoelectron spectroscopy (XPS) using a Kratos Axis Ultra-DLD (Kratos Analytical Ltd., Kyoto, Japan). Accurate binding energies (±0.1 eV) were determined regarding to the position of the C_1s_ peak. The residual pressure in the analysis chamber was maintained below 10^−9^ Torr during data acquisition and survey and multiregion spectra were recorded. Each spectral region of interest was scanned several times to obtain good signal-to-noise ratios. The atomic concentrations were calculated from photoelectron peak areas and sensitivity factors provided by the spectrometer manufacturer. The crystallinity of composites were determined by X-ray diffraction (XRD) using a Bruker D8 Advance X-ray diffractometer (BRUKER, Rivas-Vaciamadrid, Spain) (Cu Kα radiation, wavelength (λ) of 1.541 Å).

The carbonization process of composites was studied by thermogravimetric (TG) and differential thermogravimetric (DTG) analyses by heating the sample in nitrogen flow from 50 °C to 900 °C at 20 °C min^−1^ using a Mettler–Toledo TGA/DSC1 thermal balance (Mettler-Toledo International Inc., Greifensee, Switzerland). The TiO_2_ content in a given composite was estimated by ubtracting the weight loss obtained with pure TiO_2_ under air atmosphere (oxidizing conditions) until constant weight from the weight loss obtained with the composite [22].

Textural characterization of the samples was carried out by N_2_ adsorption-desorption at −196 °C with a Quantachrome Autosorb-1 apparatus (Quantachrome Instruments, FL, USA). The apparent surface area (*S*_BET_) was determined by applying the Brunauer–Emmett–Teller (BET) equation [23], while the micropore volume (*V*_micro_) and the mean micropore width (L_0_) were obtained from Dubinin–Radushkevich and Stoeckli equations, respectively [24,25]. The volume of nitrogen adsorbed at a relative pressure of 0.95 (*V*_pore_), was also obtained from the adsorption isotherms, which corresponds to the sum of the micro- and mesopore volumes according to Gurvitch’s rule [26].

The performance of materials in the photodegradation of the Orange-G (OG) dye in aqueous solutions was studied under UV irradiation. The experiments were performed using a glass photoreactor (8.5 × 20 cm) equipped with a low-pressure mercury vapor lamp (TNN 15/32, 15 W, Heraeus Headquarters, Hanau, Germany) emitting at 254 nm placed inside an inner quartz tube of 2.5 cm of diameter. The concentration of OG was determined by a UV–vis spectrophotometer (5625 Unicam Ltd., Cambridge, UK). Before catalytic experiments, all materials (800 mg) were saturated with the dye solution (800 mL) in dark to remove the adsorptive contribution. After saturation, the initial dye concentration (*C*_0_) was fitted again to 10 mg L^−1^ in all cases, and then, a UV lamp was turned on, this time being considered t = 0. Samples were taken from the reactor and centrifuged to separate the catalyst particles before analysis by the UV–vis spectrophotometer.

## 3. Results and Discussion

The closed structure of MCC required a previous solubilization with H_3_PO_4_ before Ti-impregnation. This acid treatment improved the dispersion of the Ti-active phase on the cellulose support but also, functionalized it simultaneously with different phosphorus-containing groups leading to carbon–phosphorus–Ti composites.

**Figure 1:**
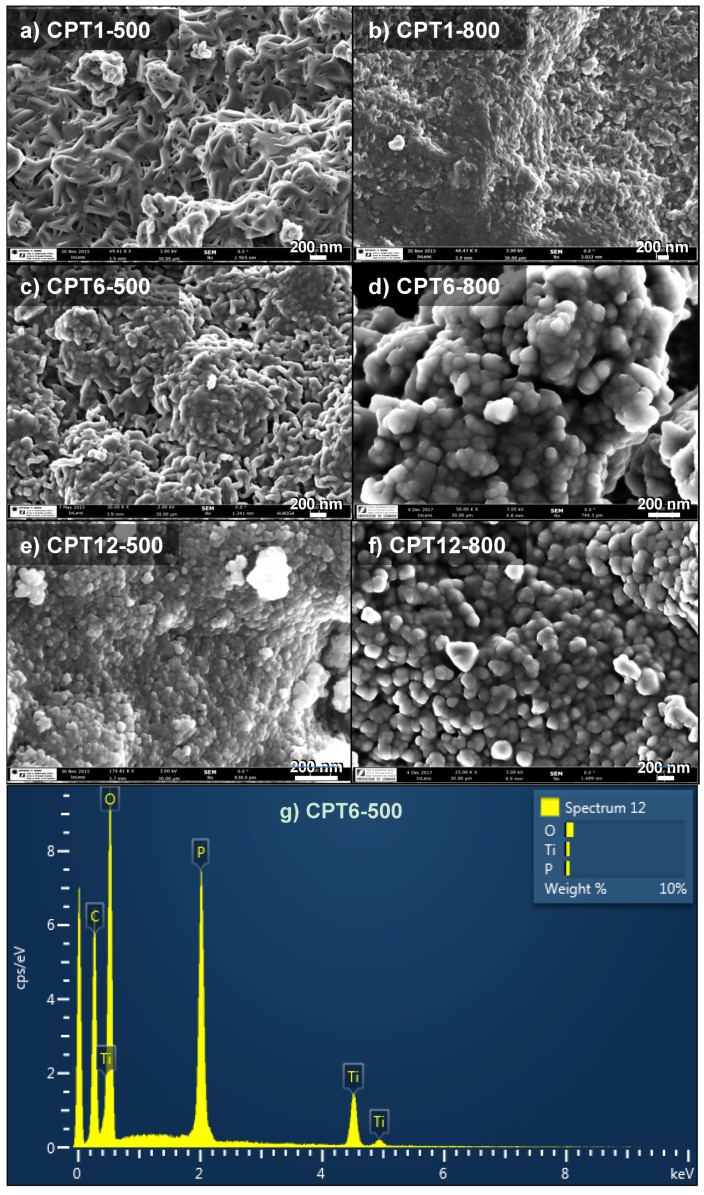
SEM micrographs for the carbon-phosphorus-Ti composites treated at 500 °C (**a**,**c**,**e**) and 800 °C (**b**,**d**,**f**), as well (**g**) EDX spectrum for the CPT6-500 composite.

The morphology of the composites was analyzed by SEM (Figure 1). The composites prepared with low and intermediate Ti:cellulose and carbonized at 500 °C, i.e., CPTi1-500 and CPT6-500, presented open structures formed by a network of elongated particles resembling the raw cellulose fibers (Figure 1a,c). These large structures become round shaped particles with increasing the Ti:cellulose ratio up to 12 wt.% (Figure 1e). After carbonization at 800 °C, round-shaped particles are observed in the surface of all samples; the particle size being increased as the Ti-content (Figure 1b,d,e). The particle size determined for the CPT12 composite after carbonization at 500 °C was always smaller than 50 nm, while some particles larger than 300 nm were detected after carbonizing at 800 °C. EDX-microanalysis of all composites showed high contents of C and Ti, but also of phosphorus (Figure 1g for CPT6-500), which was distributed homogeneously on the composite, as confirmed by EDX.

Cellulose–phosphate structures formed during the MCC solubilization with H_3_PO_4_ were reported to be reversible, i.e., they are removed after washed leading to free H_3_PO_4_ and amorphous cellulose [27]. In our case, although amorphous cellulose was obtained, the phosphorus functionalities were stable not only after being washed but also after carbonization of the composites, as corroborated below by different techniques.

Thus, the stability of the phosphorus-containing groups was confirmed by XPS. As an example, the chemical composition of the CPT6 samples and the variation on the nature of the surface groups with the carbonization temperature are summarized in Table 1. An increase of the carbonization temperature led to the progressive reduction of the samples since the oxygen content decreased (i.e., 42.7 and 36.4% for CPT6-500 and CPT6-800, respectively) due to the thermal decomposition of some oxygen and/or phosphorus functionalities, which were released as CO_x_. The deconvolution of the Ti_2p_ region showed for CPT6-500, an only peak placed at ≈459.3 eV corresponding to the presence of Ti^+4^, while the corresponding sample carbonized at 800 °C presented an additional component at ≈458.6 eV due to the presence of Ti^+3^ (Table 1). 

Analogously, a variation of the spectra of the P_2p_ region was observed for the different CPT6 samples. Thus, this region can be deconvoluted in two peaks placed at ≈132.8 and ≈133.8 eV corresponding to phosphorus linked to carbon (C-PO_3_) and to pentavalent tetracoordinated phosphorus in phosphates or polyphosphates as (C-O-PO_3_), respectively [28] (Table 1). In addition, the position of these peaks is shifted to higher binding energies (BE) with increasing the oxidation degree of the P-groups [29,30], while the peak at low BE is favored at high carbonization temperatures.

**Figure 2:**
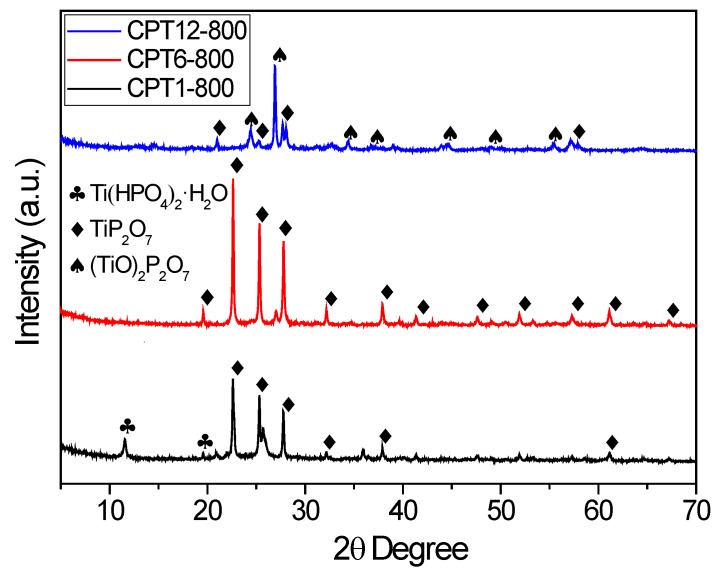
XRD patterns of the different carbon-phosphorus-Ti composites treated at 800 °C.

XRD patterns for the composites treated at 500 °C did not show any peak regardless the Ti:cellulose ratio used, denoting an amorphous character for these samples. Nevertheless, sharp peaks were observed in XRD patters when samples were treated at 800 °C, with different crystalline phases being formed depending on the Ti:cellulose ratio (Figure 2). The TiP_2_O_7_ crystalline phase (JCPDS 38-1468) was present in all these composites, but also there is a small contribution of Ti(HPO_4_)_2_ (JCPDS 38-334) at low Ti-content, i.e., CPT1-800. On the other hand, when the Ti-content is increased up to 12 wt.% (i.e., CPT12-800), the main crystalline phase was (TiO)_2_P_2_O_7_ (JCPDS 39-0207). Thus, richer crystalline Ti-phases are favored when increasing the Ti-content in the composite since the H_3_PO_4_/cellulose ratio was always maintained. The crystal size obtained by application of the Scherrer equation was 38.9, 53.4 and 57.9 nm for CPT1-800, CPT6-800 and CPT12-800, respectively.

**Figure 3:**
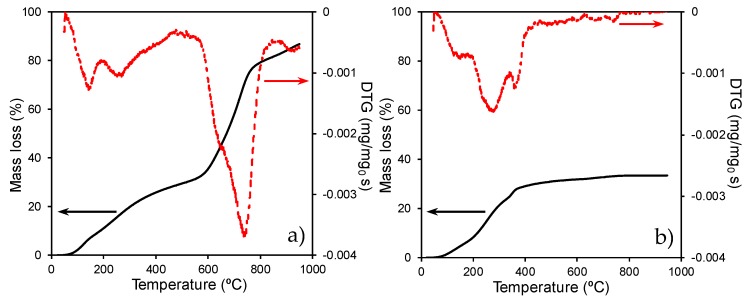
TG and DTG profiles obtained during the carbonization in N_2_ flow: (**a**) H_3_PO_4_-treated cellulose support and (**b**) CPT6 composite.

The marked influence of the carbonization temperature on the interactions of Ti-species with the phosphate surface groups was also pointed out by the thermogravimetric analysis of the samples. Thus, TG-DTG profiles obtained during the carbonization process of H_3_PO_4_-treated cellulose before (i.e., the CP support) and after Ti-impregnation (i.e., the CPT6 composite) are compared in Figure 3a,b, respectively. The support carbonization occurs in three steps denoted by the corresponding minimum in the DTG profile (Figure 3a). The first weight loss occurs at ≈120 °C and can be associated with dehydration and drying processes; a second peak at ≈240 °C corresponds to the release of CO_x_ formed by the thermal decomposition of oxygenated surface groups, namely, carboxylic acids that decompose at this temperature range [31]; and the third peak can be due to the reduction of the phosphate surface groups by the organic matrix, causing the gasification of the support, as typically described in the chemical activation process of lignocellulosic materials [32]. In the carbonization of the CPT6 composite (Figure 3b), the Ti-support interactions leads to a certain shifting of the first peaks to slightly higher temperatures compared to the support. This fact should be related with the formation of links between the oxygenated surface groups of cellulose and the Ti-species [19]. However, Ti-species mainly interact with the phosphorus-containing groups, in such a manner that the reduction of these groups by the cellulose matrix at ≈750 °C does not occur during the thermal treatment (Figure 3b), this reduction being replaced by the reaction between these groups with the Ti species leading to crystalline Ti-phosphate or polyphosphate compounds, as previously observed by XRD. In fact, the weight loss above 500 °C is clearly negligible.

**Figure 4:**
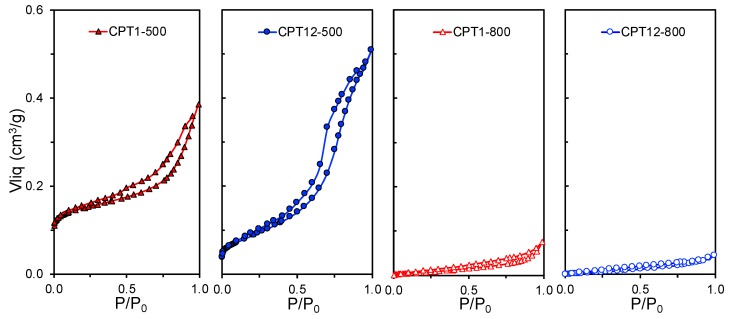
N_2_-adsorption isotherms of carbon-phosphorus-Ti composites treated at 500 or 800 °C.

The morphological and crystalline transformations of the composites previously discussed had a clear effect on their textural properties, which were determined by analyzing the corresponding N_2_-adsorption isotherms (Table 2 and Figure 4). In general, the total pore volume (*V*_pore_) and the BET surface area (*S*_BET_) of the composites decreased as the Ti-content and the carbonization temperature increased, due to the higher porosity of the carbon phase compared to inorganic Ti-phases and the sintering favored under these conditions. The CPT1-500 composite presented the highest surface area (357 m^2^ g^−1^) due to its high micropore volume (0.144 cm^3^ g^−1^) associated with a high adsorbed volume of N_2_ at low relative pressure (Figure 4). In general, the isotherms of the composites belong to type-IV or type-II, showing from P/P_0_ >0.4 a clear hysteresis cycle due to the presence of mesopores. A similar behavior is also observed for CPT6 and CPTi12 samples with a loss of microporosity but also an enhanced mesoporosity (i.e., higher adsorbed volume of N_2_ at high relative pressure), compared to composites prepared with lower Ti-content.

**Figure 5:**
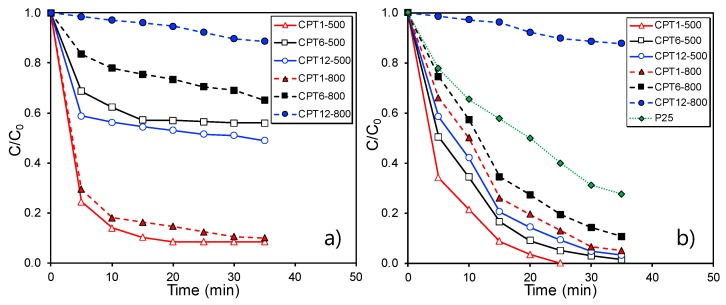
Removal of the Orange-G from water solution by adsorption (**a**) and photocatalytic (**b**) processes using carbon-phosphorus-Ti composites.

The adsorptive and photocatalytic performance of the carbon–phosphorus–Ti composites were analyzed for the removal of OG (Figure 5a,b, respectively). Firstly, all samples were saturated in dark experiments, which hinders the contribution of the adsorption process to the OG removal in the subsequent photocatalytic experiments. The adsorption capacity and the adsorption rate are not exclusively related to the different porosity of the samples, as observed in Figure 5a. In general, composites with lower Ti-content presented a better adsorptive behavior than those prepared with intermediate and high Ti-contents regardless of the carbonization temperature used. The maximum removal of OG was achieved after 20 min and varied as: CPT1 samples > CPT6 samples > CPT12 samples. This trend could be explained because composites with low Ti:carbon ratio present a larger carbon phase, which has a higher affinity for OG in solution. The CPT1 composites presented the best adsorptive behavior, being the adsorption of both CPT1-500 and CPT1-800 comparable in spite of their different porous characteristics and the crystallinity of their Ti-phases.

In Figure 5b, we show the photocatalytic efficiency obtained for the different carbon–phosphorus–Ti composites and the benchmark TiO_2_ material (Degussa P25) for comparison. The complete OG removal was achieved after ≈25–35 min or 40–50 min depending on composites treated at 500 or 800 °C, respectively. In general, all composites obtained at low carbonization temperature presented a better efficiency than those treated at 800 °C; in spite of that, all these samples were completely amorphous since no peaks were observed by XRD. The carbon phase retards the crystal growth and the phase transformations of metal oxides in carbon–metallic oxide composites [33,34]. In addition, the presence of phosphorus may influence the TiO_2_-crystal structure, obtaining mixtures of TiO_2_ amorphous and anatase [35]. In this context, composites obtained at 500 °C could develop a mixture of amorphous and very small TiO_2_-anatase nanoparticles (undetectable by XRD), with the latter being responsible for the high activity of composites treated at 500 °C. On the other hand, the photocatalytic efficiency varied as follows: CPT1-500 > CPT6-500 > CPT12-500, i.e., when the Ti-content increased in the composites, which could be related with their lower porosity (Table 2).

Concerning composites treated at 800 °C, the formation of different polyphosphates was pointed out by XRD. The band gap for the titanium pyrophosphate (TiP_2_O_7_) was estimated to be 3.48 eV [36], with is higher than that for the TiO_2_ anatase or rutile phases, i.e., 3.2 and 3.0 eV, respectively. The different nature of polyphosphates can also influence their performance [37,38].

In addition, sintering was favored to this range of temperature, with Ti-particles larger than ≈39 nm being obtained. Otherwise, even the composites carbonized at this temperature, with exception of CPTi12-800, presented a better performance than the benchmark TiO_2_ material (Degussa P25). This fact denotes the importance of the carbon phase in Ti-based composites, leading to an enhanced improved performance based on the synergism between both phases. Overall, carbon–phosphorus–titanium composites with low carbon content and carbonization temperature are preferred for the removal of the OG pollutant by photocatalysis because of their enhanced porosity, high dispersion of the active phase (anatase) and strong adsorption capacity (interaction) of OG.

## 4. Conclusions

The treatment of microcrystalline cellulose with H_3_PO_4_ leads to a simultaneous functionalization of cellulose chains by incorporating stable phosphorus-containing surface groups, namely, phosphates and polyphosphates. These functionalities interact and react progressively with Ti-species during impregnation and carbonization at high temperatures, with different polyphosphates of titanium being anchored on the carbon phase. The physicochemical properties of these carbon–phosphorus–Ti composites vary according to the Ti-content and carbonization temperature. Thus, the increase of these parameters favors Ti-particle sintering, the formation of Ti-crystalline phases and a marked loss of the porosity. The synergism between phases allows to obtain materials with enhanced photocatalytic efficiency compared to the benchmark TiO_2_ material (Degussa P25), in spite of the band gap of polyphosphates being wider than that for anatase/rutile phases. Carbon–phosphorus–Ti composites with anatase TiO_2_ nanoparticles and large surface areas seem to be the most active photocatalysts for OG degradation under UV irradiation.

## Figures and Tables

**Table 1:** Surface concentration, species percentage and corresponding binding energies (in brackets, eV) obtained for the CTP6 sample obtained at different carbonization temperatures.

Sample	C	O	P	Ti	P_2p_ (%)		Ti_2p_ (%)	
	(wt.%)				C-PO_3_	C-O-PO_3_	Ti^3+^	Ti^4+^
CPT6-500	22.0	42.7	21.9	13.4	36 (132.9)	64 (133.8)	-	100 (459.3)
CPT6-800	27.3	36.4	22.8	13.5	63 (132.8)	37 (133.8)	48 (458.6)	52 (459.5)

**Table 2:** Textural properties of selected carbon-phosphorus-Ti composites treated at 500 or 800 °C.

Sample	*S*_BET_ (m^2^ g^−1^)	*V*_micro_ (cm^3^ g^−1^)	*V*_pore_ (cm^3^ g^−1^)
CPT1-500	357	0.144	0.386
CPT6-500	28	0.013	0.160
CPT1-800	9	0.004	0.076
CPT12-500	184	0.073	0.508
CPT6-500	30	0.017	0.239
CPT12-800	5	0.021	0.043
